# Application of artificial intelligence in diagnosis and treatment of colorectal cancer: A novel Prospect

**DOI:** 10.3389/fmed.2023.1128084

**Published:** 2023-03-08

**Authors:** Zugang Yin, Chenhui Yao, Limin Zhang, Shaohua Qi

**Affiliations:** ^1^Department of General Surgery, The First Affiliated Hospital of Dalian Medical University, Dalian, China; ^2^Department of Respiratory, The First Affiliated Hospital of Dalian Medical University, Dalian, China; ^3^Institute of Laboratory Animal Science, Chinese Academy of Medical Sciences and Comparative Medicine Center, Peking Union Medical College, Beijing, China

**Keywords:** artificial intelligence, colorectal cancer, machine learning, deep learning, bioinformatics analysis, screening, diagnosis, therapy

## Abstract

In the past few decades, according to the rapid development of information technology, artificial intelligence (AI) has also made significant progress in the medical field. Colorectal cancer (CRC) is the third most diagnosed cancer worldwide, and its incidence and mortality rates are increasing yearly, especially in developing countries. This article reviews the latest progress in AI in diagnosing and treating CRC based on a systematic collection of previous literature. Most CRCs transform from polyp mutations. The computer-aided detection systems can significantly improve the polyp and adenoma detection rate by early colonoscopy screening, thereby lowering the possibility of mutating into CRC. Machine learning and bioinformatics analysis can help screen and identify more CRC biomarkers to provide the basis for non-invasive screening. The Convolutional neural networks can assist in reading histopathologic tissue images, reducing the experience difference among doctors. Various studies have shown that AI-based high-level auxiliary diagnostic systems can significantly improve the readability of medical images and help clinicians make more accurate diagnostic and therapeutic decisions. Moreover, Robotic surgery systems such as da Vinci have been more and more commonly used to treat CRC patients, according to their precise operating performance. The application of AI in neoadjuvant chemoradiotherapy has further improved the treatment and efficacy evaluation of CRC. In addition, AI represented by deep learning in gene sequencing research offers a new treatment option. All of these things have seen that AI has a promising prospect in the era of precision medicine.

## 1. Introduction

**Figure 1:**
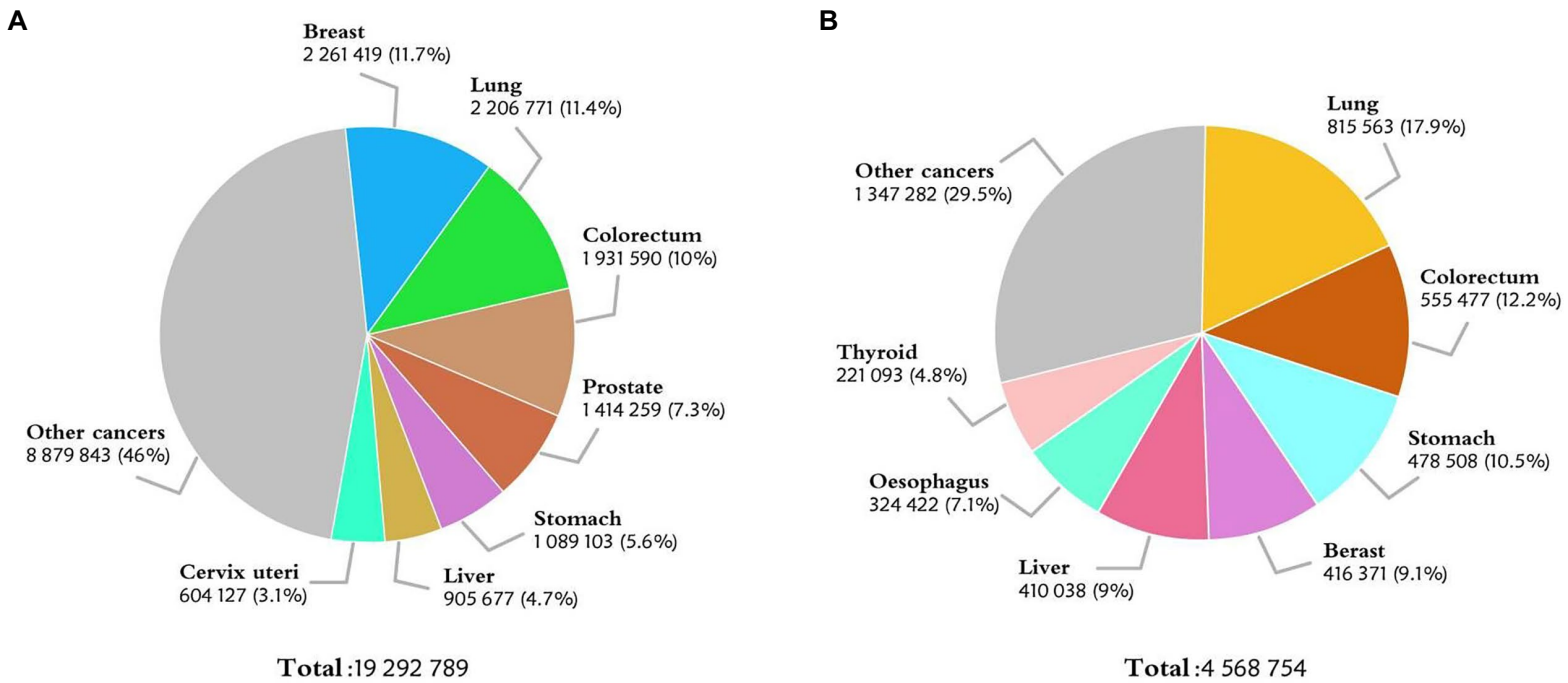
**(A)** Estimated number of new cases in 2020, World, both sexes, all ages. **(B)** Estimated number of new cases in 2020, China, both sexes, all ages. (Data source: GLOBOCAN 2020).

Colorectal cancer (CRC) is a common disease that threatens the public health. According to the International Agency for Research on Cancer, there were an estimated 1.93 million new cases of CRC worldwide in 2020, making itself in the third place in the most common cancer list (Figure 1A). The incidence of CRC is particularly significant in countries undergoing social and economic transition. In China, there were about 560 thousand newly diagnosed cases of CRC in 2020, second only to lung cancer in terms of morbidity (1) (Figure 1B). Based on the GLOBOCAN 2020 cancer assessment and population data from the World Health Organization (WHO), the number of new cases of CRC in China is estimated to reach 590 thousand in 2022, more than any other countries in the world (2). Besides smoking, obesity, and unhealthy lifestyle, the incidence of CRC is also related to gender, genetic cause, and family factors (3–6). Currently, the main diagnostic methods for CRC include laboratory tests, endoscopy, imaging and histopathology examination, etc. Traditional ways of treating CRC entail surgery, radiotherapy, and post-metastasis therapy, among others (7–10). Despite all these tools, the rise in CRC incidence and mortality is alarming. With the recent attention of early screening and the rapid development of precision medicine (4, 11), a new diagnosis and treatment model for CRC is on the horizon.

**Figure 2:**
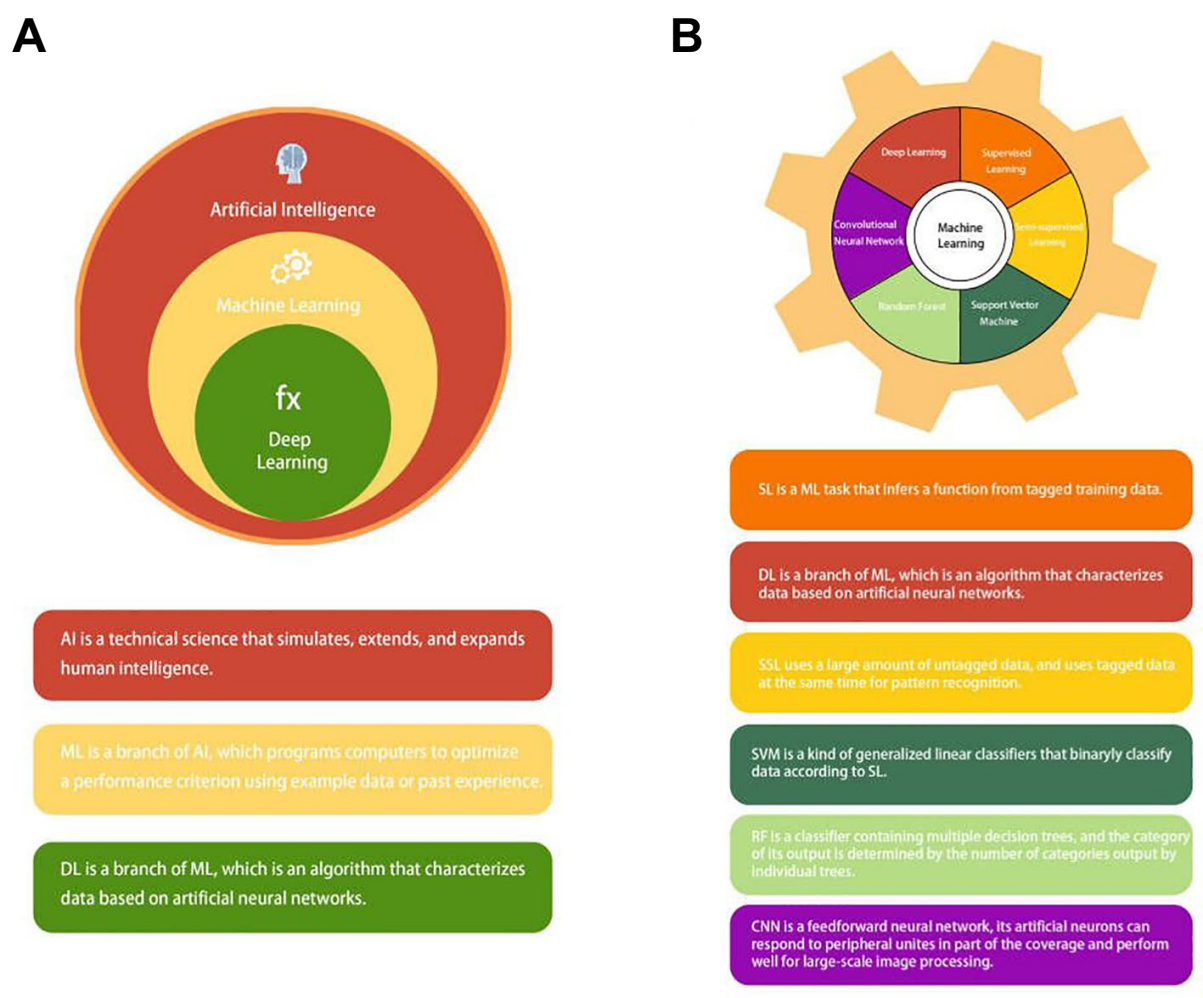
**(A)** The concept and relationship of Artificial intelligence (AI), machine learning (ML), and deep learning (DL). **(B)** Common types of machine learning (ML): supervised learning (SL); deep learning (DL); semi-supervised learning (SSL); support vector machine (SVM); random forest (RF); and convolutional neural network (CNN).

Artificial intelligence (AI) can be understood as studying the principle of human intelligence activities, constructing an artificial system with certain intelligence, and studying how to let computers complete the work that requires human intelligence in the past. Nowadays, with the rapid development of computer technology and the vigorous promotion of precision medicine, the application of AI in medicine is also in full swing (12, 13). AI applications in medicine are now divided into virtual and physical branches (12). Machine learning (ML) is an essential subbranch of AI. It also can be divided into subsets such as deep learning (DL), supervised learning (SL), semi-supervised learning (SSL), support vector machine (SVM), random forest (RF), and convolutional neural network (CNN) (14–17) (Figure 2). Among them, DL and CNN are representatives of the most successful algorithms used in medicine in recent years (17, 18). They play a very broad role in data management (19), information control (20), diagnosis prediction (21), and drug delivery (22). The branches of physics mainly include medical equipment (23) and robot applications, such as da Vinci robot system, which is widely used today (24, 25). This article mainly discusses the development of AI and its application in the diagnosis and treatment of CRC.

## 2. The development of AI

It is generally believed that the development of AI evolved from robots. The word “robot” first appeared in the works of the Czech dramatist Karel Capek in the early 20th century, referring to forced labor or compulsory work (26). However, the topic about robots has been popular in Chinese and Western cultures for a long time. Hephaestus and his robotic dogs constantly featured in ancient Greek and Roman myths. Aristotle’s genius prediction for robots in his Politics also reflected people’s beautiful vision of robots at that time. There are also many stories about automata in ancient China. More than 3,000 years ago, a mechanic named Yan Shi presented King Mu of Zhou to a human-size mechanical device. To drink and recite poems with Liu every day, Emperor Yang of the Sui Dynasty ordered the craftsman to create a wooden mechanical man according to Liu’s figure. This wooden man could kneel and toast like Liu (12, 26, 27). Leonardo da Vinci also made a major contributor to medical physics. He presented his invention of automata robots, a mobile knight, in 1495. In the da Vinci notebook, we found details and sketches of manufacturing robots. With the approval of the FDA, the first generation da Vinci surgical system was manufactured and put on the market in 1999. So far, robot-assisted surgery has been widely used (12, 26–28).

In 1950, Alan Turing experimentally detected some machines that showed intelligent behavior like human, which we call the “Turing test.” At the Dartmouth conference in 1956, John McCarthy and his team officially proposed the concept of AI (13, 29–31). Over the past 60 years, AI has made great progress in all walks of life. AI has apparent advantages in solving complex nonlinear parameter problems, and its accurate prediction ability has made it widely used in waste generation, collection, management, and conversion processes (32). AI is also considered to play an important role in addressing rising demand, road forecasting, planning, and management, self-driving and safety (33, 34). The development of educational AI can reduce the expenditure budget and the burden on teachers and provide more personalized teaching services for each student. On the other hand, it can also help realize educational opportunities for more students (35). Over the past few years, the AI player represented by AlphaGo has defeated the world Go champions, including Jie Ke, indicating that we have made exciting progress in the computer Go. These results are primarily based on the creative combination of deep convolutional neural networks (DCNN) and Monte Carlo tree search (MCTS). We still need to further understand the working mechanism of the model through the visual operation (36). AI can also have an impact on monitoring and controlling the spread of the virus. Vaishya et al. have obtained a result-driving technology through experiments, which can play a role in the early screening of COVID-19, the detection and tracking of infected patients, the formulation of adjuvant treatment plans, as well as the development of vaccines (37).

With the development of ML and DL, AI has become more and more widely used in medicine and has bright prospects in disease prediction, treatment, and prevention (29, 38, 39). This article mainly reviews the diagnosis and treatment of AI in CRC.

## 3. The application of AI in CRC diagnosis

**Figure 3:**
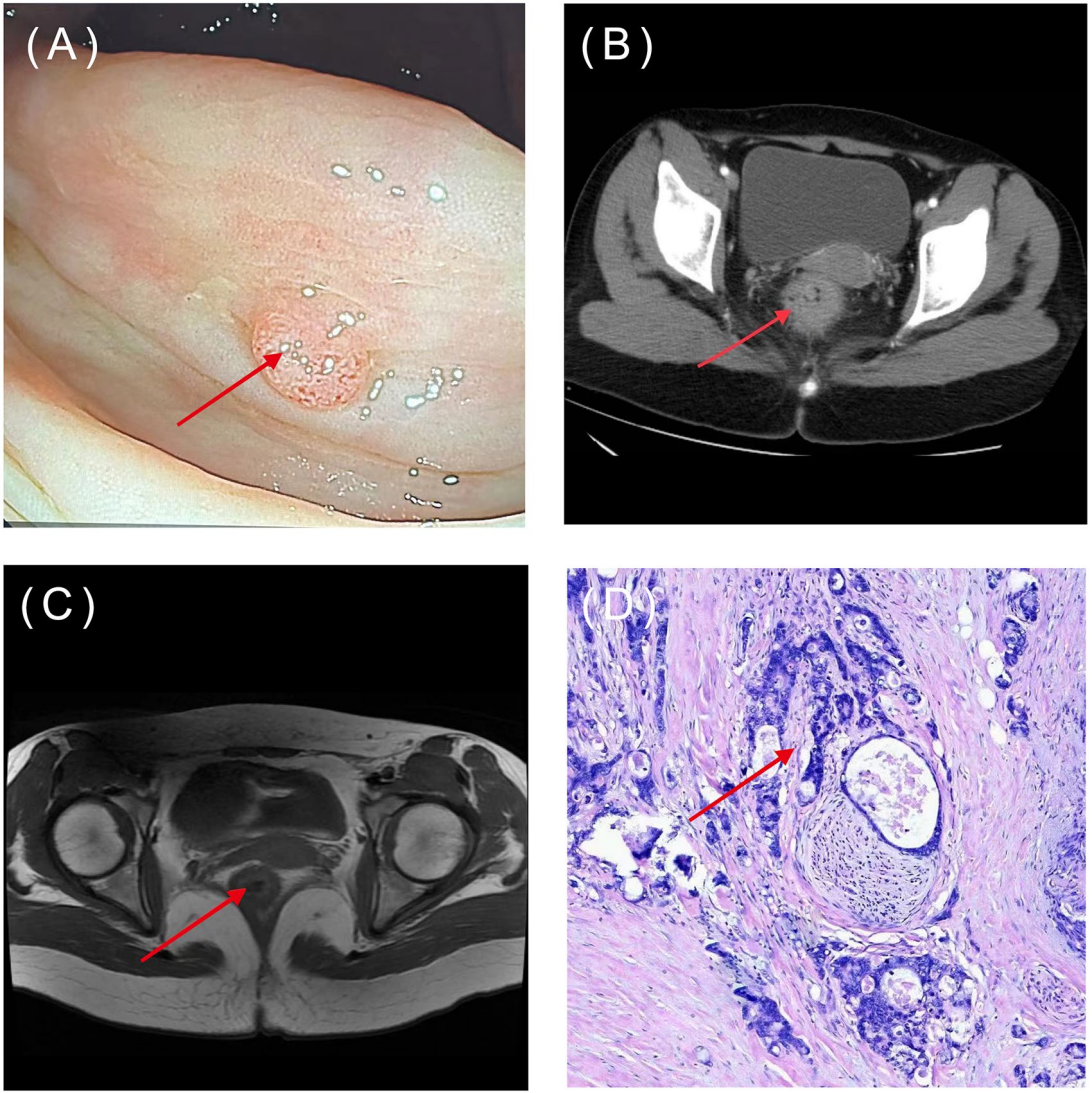
Common types of CRC diagnostic images: **(A)** Endoscopy; **(B)** CT; **(C)** MRI; **(D)** pathology image (HE×100). The arrow indicates the location of the lesions. (Image source: The First Affiliated Hospital of Dalian Medical University.)

The application of AI in CRC screening can improve the early screening rate and thus significantly reduce the incidence and mortality of CRC patients. The bioinformatics tools embedded in AI can help screen and identify more CRC biomarkers. AI assisted pathology recognition technology can help pathologists improve efficiency, reduce workload, and lower the rate of misdiagnosis and missed diagnosis. ML, widely used in AI image recognition, can greatly improve the readability of medical images, reduce empirical errors, objectively provide reliable and comprehensive reference opinions, and help doctors make more accurate clinical decisions (Figure 3). Common AI models for CRC diagnosis are summarized in Table 1.

**Table 1:** The summary of the application of AI in the diagnosis of CRC.

Theme	Year	Subject	Model	Sample	Result	Ref
Endoscopic diagnosis	2018	Polyp detection	CNN	155 videos	AUC = 0.87	(40)
	2019	Neoplasia detection	CNN	685 subjects	ADR = 54.8%	(41)
	2020	Adenoma detection	DL	386 patients	AMR = 13.89%	(42)
	2022	Neoplasia detection	CNN	230 subjects	AMR = 15.5%	(43)
	2020	Polyp and adenoma detection	CNN	308 patients	ADR = 0.289;0.367(polyp; adenoma)	(44)
	2020	Polyp detection	YOLO	150 patients	PDR = 38.7%	(45)
	2022	Polyp and adenoma detection	CNN	1,434 patients	PDR = 40.8% ADR = 20.1%	(46)
	2021	Adenoma detection	CADe	1,076 patients	ADR = 21.27%	(47)
	2021	Polyp	DL	2,352 patients detection	PDR = 38.8%	(48)
	2019	Polyp and adenoma detection	DL	522 patients	ADR = 29.1% PDR = 64.93%	(49)
	2021	Adenoma detection	CNN	358 patients	AMR = 13.8%	(50)
Non-invasive screening	2022	Cancer diagnosis and stage	RF/SVM/DT	521 samples	Average accuracy = 99.81% F1 value = 0.9968 accuracy = 99.88%recall = 99.5%	(51)
	2021	Biomarkers screening	SVM/LR/RF/kNN/NB	1,164 electronic medical records	AUC = 0.849	(52)
	2019	Cancer detection	LR/SVM	817 plasma samples	Mean AUC = 0.92 mean sensitivity = 85% specificity = 85%	(53)
	2020	Cancer screening	ML	289 healthy individuals and 983 patients	Specificity = 0.89 sensitivity = 0.72	(54)
	2019	Mutation detection	CP-ANN	312 tissue samples	Sensitivity = 100% specificity = 87.5% accuracy = 93.8%	(55)
	2020	Gene detection	DL	8,836 samples	Mean AUROC = 0.92 AUPRC = 0.63	(56)
	2020	Gene identification	LASSO	480 CRC and 41 normal tissues	AUC = 0.6923 (training set; 3-year) AUC = 0.7328 (training set; 5-year) AUC = 0.6803 (testing set; 3-year) AUC = 0.7035 (testing set; 5-year)	(57)
	2021	Gene detection	CGANs	256 patients (training cohort 1) 1457 patients (training cohort 2)	AUROC = 0.742 (training cohort 1) AUROC = 0.757 (training cohort 2) AUROC = 0.743 (synthetic data) AUROC = 0.777 (mixed data)	(58)
Histopathologic diagnosis	2021	Image learning	DELR	500–3,000 samples	AUC > 0.95	(59)
	2022	Histopathologic segmentation	CNN/TL	25 WSIs	DSI = 82.74% ± 1.77 accuracy = 87.07% ± 1.56 f1-score value = 82.79% ± 1.79	(60)
	2021	Distinguish CRLM	DL/CNN/ICC	93 CRLM patients	AUC = 0.69	(61)
	2020	Histopathologic classification	CNN/RNN	4,036 WSIs	AUC = 0.96; 0.99(adenocarcinoma; adenoma)	(62)
	2021	Histopathologic segmentation	PCA/DWT	351 specimens	Dice = 0.804 ± 0.125	(63)
	2021	Image classification	ANN/SVM	5,000 histopathology image tiles	Performance accuracy = 95.3%	(64)
	2022	Histopathologic screening	DL/ML	294 WSIs	AUC = 0.917 sensitivity = 97.4%	(65)
	2022	Histopathologic classification	DL/CNN	1,865 pathological images	AUC = 0.995; 0.998	(66)
	2022	Image grading	CNN/HCCANet	630 images	Overall accuracy = 87.3% average AUC = 0.9	(67)
	2019	Survival prediction	TL/CNN	862 HE images	Accuracy>94%	(68)
	2019	Cancer diagnosis	CNN/RE/kNN/LR/NB/SVM	357 images	Accuracy = 87–95%	(69)
Radiologic diagnosis	2019	Preoperative Assessment	MLP/LR/SCM/DT/RF/KNN	3T-MRI imaging from 152 patients	AUC = 0.809; 0.746 sensitivity = 76.2%; 79.3% specificity = 74.1%; 72.2% (MLP; RF)	(70)
	2021	Cancer response prediction	ML	MRI scanning from 72 patients	AUC = 0.793	(71)
	2021	Image segmentation	U-Net	T2WI segmentation from 300 LARC patients	Mean DSC = 0.675 median DSC = 0.702	(72)
	2020	RC Circumferential Evaluation	Faster R-CNN	detect 12,258 T2WIs	Accuracy = 0.932 sensitivity = 0.838 specificity = 0.956	(73)
	2022	CRCLM early diagnosis	FM	CT scan from 30 patients	Precision = 100% overall accuracy = 93.3% recall = 77.8%	(74)
	2021	Tissue assessment	ResNet	OCT differentiate from 43, 968 cancer and 41, 639 norm ROIs	AUC = 0.975	(75)
	2021	Diagnosis detection	DLLD	4,386 CT images from 502 patients	Sensitivity = 81.82% false positives = 1.330	(76)
	2021	Metastasis prediction	DLRS	Collect and predict from 235 nCRT patients	AUC = 0.894	(77)
	2019	Accurate segmentation	LAGAN	CT scan and segment from 223 CRC patients	DSC = 90.82%; 91.54% (FCN32; U-Net)	(78)
	2020	Metastasis prediction	ResNet	CT scan from 192 CRLM patients	AUC = 0.903	(79)
	2022	Cancer segmentation	U-Net/CNN	Analysis 201 MRI images	DSC = 0.727; 0.930; 0.917 (tumor; rectum; mesorectum)	(80)
	2020	Predict response	DL	T2W MRI predict 383 participants	AUC = 0.99	(81)
	2021	Detect differentiation	RF	169 CT images segmentation from 63 patients	AUC = 0.91 sensitivity = 82% specificity = 85%	(82)
	2021	Improve prognostication	RF	MRI identifies 94 lesions from 55 patients	AUC = 0.94	(83)
	2020	Real-time diagnosis	DL	26,000 OCT images	AUC = 0.998	(84)

ADR, adenoma detection rate; AUC, area under the curve; AMR, adenoma miss rate; AUPRC, area under the precision-recall curve; AUROC, area under the receiver operating characteristic; ANN, artificial neural network; CADe, computer-aided detection; CRCLM, colorectal cancer liver metastasis; CNN, convolutional neural network; CGANs, conditional generative adversarial networks; CRC, colorectal cancer; CP-ANN, counter propagation artificial neural network; DL, deep learning; DSI, dice similarity index; DWT, discrete wavelet transform; DELR, deep embedded-based logical regression; DLLD, deep learning based lesion detection algorithm; DLRS, deep leaning radiomic signature; DSC, dices similarity coefficient; DT, decision trees; FCN, fully conventional network; FM, Formal Methods; ICC, intra-class correlation coefficient; kNN, nearest neighbors; LR, logical regression; LARC, local advanced rectal cancer; LASSO, least absolute shrinkage and section operator; LAGAN, label assignment generative adverbial network; ML, machine learning; MSI, microsatellite instability; NB, naive baves; OCT, optical coherence tomography; PCA, principal component analysis; PDR, polyp detection rate; RNN, recurrent neural networks; RC, rectal cancer; RF, random forest; ResNet, residual network; SVM, support vector machine; TL, transfer leaning; TWI, T2-weighted images; U-net, U-shaped neutral network; WSI, whole slide images.

### 3.1. Endoscopic diagnosis

Colonoscopy has long been regarded as the gold standard procedure for diagnosing colorectal diseases, and it is strongly recommended as an early screening criterion by national associations (85). Due to the high operator variability of quality, challenging and frequently inadequate preparation, high loss of work productivity, and so on, the detection rate of polyps and adenomas in early colorectal screening often varies greatly (86).

**Figure 4:**
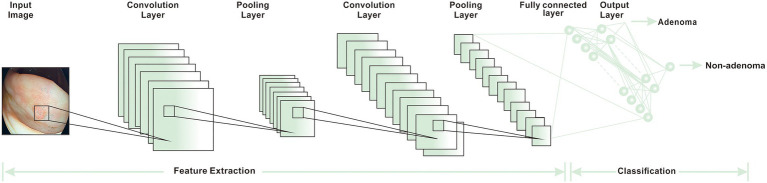
The basic workflow of CNN (15).

Researchers have used computer-aided detection (CADe) systems and AI, based primarily on DL algorithms, to improve the speed and accuracy of clinical detection of CRC and reduce the detection of missed lesions (50). The adenoma detection rate (ADR) is a reliable indicator for CRC detection. A higher ADR is often linked to a lower incidence and mortality in CRC patients (87). The combination of AI and colonoscopy can effectively improve ADR. It not only reduces the risk of CRC but also achieves the purpose of accurate resection, avoiding excessive burden on clinical work caused by the resection of many non-neoplastic polyps. A YOLOV3 AI algorithm was utilized to detect real-time polyps *via* media. It could accomplish an excellent effect in a short time. It was economical and affordable, making it suited for large-scale promotions in underdeveloped areas (88). Some adenomas and polyps detected by the real-time CADe system are small and low risk. Such adenomas and polyps are also easily ignored by endoscopists using traditional colonoscopy. Therefore this CADe system increases the incidence of CRC to a certain extent (49). A large number of studies are supporting AI-assisted colonoscopy in the diagnosis of CRC. Some optical biopsy techniques, which can capture real-time images manually, have also been proved to have a lot of potential in medical applications (45, 89). Another widely recognized colonoscopy index is adenoma miss rate (AMR), which refers to the difference between the lesions detected by consecutive endoscopy. Kamba et al. established a CADe system that uses the CNN algorithm to aid in the detection of AMR. The result demonstrated that the AMR of the CADe-assisted group was 22.9%, which was lower than that of the standard colonoscopy group. At the same time, the difference in ADR between the two groups was also only 10.9% (Figure 4). These results showed that AMR has greatly decreased with the assistance of AI. The AMR was more sensitive in detecting lesions than ADR indicators (50).

In addition, after the colorectal polyps are screened out, it is also very necessary to identify benign and malignant polyps. As endoscopic tools, including magnifying endoscopy, chromoendoscopy, confocal laser endomicroscopy, and autofluorescence endoscopy, continue to advance, the combination of AI and colorectal endoscopy has injected new impetus for future diagnosis (90). Japanese experts previously used narrow-band imaging (NBI) to classify magnifying endoscopes. They were earlier to apply NBI imaging technology and magnifying endoscopes in the clinical application (91). By combining NBI with magnification endoscopy, Gonai et al. calculated the difference between microvessel density in colorectal lesions, which was used as the differential point for the diagnosis of CRC and adenoma (92). In the same year, some experts in the United States developed CADe algorithms to distinguish tumor polyps and non-tumor polyps based on probe confocal laser endoscopy. The sensitivity, specificity and sensitivity are more than 90% (93). It is expected that the use of AI can greatly improve the accuracy of endoscopic diagnosis, and reduce the misdiagnosis rate and overtreatment.

### 3.2. Non-invasive screening

The main form of non-invasive screening is the detection of various relatively specific tumor markers from ascites, feces, blood, and other samples. Compared with colonoscopy screening, the non-invasive screening is not only lower risk, but it’s preparation time is shorter (94). However, there are few effective tumor markers for the detection of early CRC. Common non-invasive screening methods such as fecal occult blood test (FOBT) and carcinoembryonic antigen (CEA) have low sensitivity and specificity (95–97). In recent years, ML has been widely used in medical data analysis, which can help us improve the accuracy of existing biomarkers and screen out more potential marker genes (51).

CEA is one of the most studied colorectal tumor markers, but the screening of serum CEA has limited sensitivity in asymptomatic people (97). Li et al. extracted some of the most common markers from the laboratory blood tests and used five ML models to identify CRC patients from healthy people. The results showed that the logistic regression model could greatly improve the sensitivity and specificity of CEA. The model was an effective, economical, and non-invasive method for CRC identification (52). Common genes used for CRC DNA mutation detection include BRAF and KRAS, but these tumor markers are not sensitive or specific enough to show CRC (98). In 2019, Zhang et al. used near-infrared (NIR) spectroscopy combined with counter propagation artificial neural network (CP-ANN) to distinguish a BRAF V600E mutant from wild-type samples in CRC tissues. This method had a sensitivity of 100% and specificity of 87.5% for detecting the BRAF V600E mutant in CRC. It could be used for the auxiliary diagnosis of the BRAF mutation in CRC (55). The detection of abnormal DNA methylation markers in plasma or feces is a promising approach for the non-invasive early diagnosis of CRC. The SEPT9 gene methylation test has been used commercially as an alternative for CRC screening (94, 99, 100). However, the specificity of detection methods based on single DNA methylation sites is limited (101). In 2019, Kel et al. used a method called “Walking away” to collect data samples from 300 CRC patients, analyzing the data by ML and bioinformatics methods. They ultimately selected six DNA methylation epigenetic biomarkers for optimal cancer detection potential (102). The combined detection of abnormal DNA methylation markers can improve the detection rate of CRC.

Bioinformatics tools are increasingly used to analyze the pathogenesis of cancer. The effective combination of bioinformatics analysis and ML can screen and identify early CRC biomarkers, playing a role in evaluating the prognosis of CRC patients (51, 103). Hammad et al. recently identified 105 differentially expressed genes (DEGs) and 10 hub genes through bioinformatics analysis of gene expression microarray data in the Gene Expression Omnibus (GEO) database. The researchers used these tools, including SVM, Receiver operating characteristic curve (ROC), and survival analyses, to predict the diagnostic value of hub genes as CRC biomarkers. The results showed that the area under ROC curve (AUC) values of all genes were more than 0.92, confirming that these genes are expected to be biomarkers for CRC (98) With the development of sequencing technology, many non-coding RNAs (ncRNAs) have been discovered. The ncRNAs include messenger RNAs (mRNAs), microRNA (miRNAs), long non-coding RNAs (lncRNAs) and so on. Their extracellular properties are stable, easy to extract and preserve, and thus they can be studied in different body fluids (54, 104–106). MicroRNAs (miRNAs) are a class of endogenous ncRNAs with a length of about 22 nucleotides. They have a variety of important regulatory functions in the cell and are associated with the development and metastasis of cancer. There is increasing evidence that miRNAs are potential biomarkers of CRC (105, 106). In 2019, Zhang et al. identified whether miR-31 could be used as a biomarker for the diagnosis of CRC lymph node metastasis (LNM) through bioinformatics analysis techniques, including Kyoto Encyclopedia of Genes and Genomes (KEGG) enrichment analysis, correlation analysis, survival analysis, validation of expression levels, protein–protein interactive (PPI) network construction, and Gene ontology (GO). These researchers found that miR-31 was significantly increased in the plasma and tissue of CRC patients with LNM. They predicted that TNS1 might be a targeted protein for miR-31, which has an important prognostic value for patients (105). Wang et al. also found that the expression level of miR-1-3p was down-regulated in CRC by the bioinformatics analysis, suggesting that the miR-1-3p may have the potential to diagnose CRC and inhibit tumor cells progression (106).

### 3.3. Histopathologic diagnosis

Pathology is the gold standard for tumor diagnosis. It can identify the tumor cell types, stage the tumor and guide the treatment plan of patients. Meanwhile, it can also be used as a prognosis and tumor recurrence predictor. Now, a lot of diagnostic work still needs to be completed by pathologists alone. With the continuous breakthrough of digital pathology (DP) technology, it is expected to become the development direction of pathology in the future. DP can be used in image retrieval, pattern recognition, ML, and DL. By extracting corresponding quantitative features or identifying specific regions of interest (ROI), DP can build AI computer-aided diagnostic system with automatic recognition functions. Many studies have proved that the application of AI technology can help pathologists improve diagnosis efficiency, reduce workload, and improve the working environment, increasing diagnosis rate and reducing misdiagnosis rate (107). Kasahara et al. used AI to collect 146 T1 CRC cases. They analyzed the nuclear morphological characteristics in hematoxylin and eosin (HE)-stained slide images. The results suggested that the model could increase the accuracy of preoperative lymph node metastasis prediction, which needs to be further verified in clinical practice (108).

One of the most successful examples of DL’s widespread application in medicine is CNN, which has virtually reinvented image analysis technology. In a retrospective study, researchers obtained more than 100,000 HE image patches from 86 CRC tissue slides, and they used these images to train CNN based on transfer learning (TL). The results showed a nine-class accuracy of over 94% in 7180 independent data sets of 25 CRC patients. It does confirm that CNN can separate histological images and predict the survival rate of CRC patients after treatment (68). High-intensive workload and accuracy requirement for reading pathological images sometimes lead to the misdiagnosis of pathologists. Wang et al. proposed a CNN-based method to classify a large number of histopathological images. The AUC of this model was up to 0.988, with the ability to distinguish CRC from other benign tissues (18). Furthermore, by using CNN, some researchers proposed a clinically comparable technology. This model could be used to stage tumor and classify HE-stained colon histopathological images. They confirmed that the model’s classification could reach more than 90% equally by processing four different data sets (109).

Nowadays, most AI-assisted pathology recognition technologies are achieved by SL. In order to address the problem of massive data markers in SL, an SSL based on the average teacher structure was proposed. A series of expansion experiments also confirmed that SSL significantly reduces the amount of impractical labeled data and expands the fundamental reality of AI in medical work. SSL achieved the comparable effect as SL with less labeled data (110). More interestingly, an monogram model combined with ML-pathomics, immunoscore, radiomics, and clinical factors, was proved to effectively predict the postoperative prognosis of patients with CRC lung metastasis (111). These indicate significant progress in AI-assisted pathological reading. The AI-assisted pathology recognition is expected to break the current limitations that are only applied to the primary screening stage, providing more guidance and decision-making for treatment and prognosis of the CRC patients.

### 3.4. Radiologic diagnosis

Radiomics refer to a technology that converts medical images into high-dimensional available data for cancer diagnosis and prognosis (112). At present, Methods commonly used for CRC imaging evaluation include MRI, CT, and ultrasonography. Conventional imaging evaluation methods have certain shortcomings, such as limited local tumor evaluation, low tumor staging accuracy, and excessive reliance on the clinical diagnose of imaging physicians (113). Although functional magnetic resonance including diffusion-weighted imaging (DWI), T2-weighted imaging (T2WI) shows high predictive performance (112, 114). AI systems will learn to extract and integrate a large amount of imaging information to achieve a more accurate diagnosis.

The combination of AI and radiomics can extract information from various imaging data. It can be used for tumor segmentation, feature extraction, and the model establishment, eventually achieving the purpose of tumor quantitative evaluation. It has gradually become a crucial component of CRC precision diagnosis and treatment. In 2018, Liu et al. proposed a label assignment generative adversarial network (Lagan), and accurately segmented ROI in the analysis and diagnosis of CRC with CT. The application of this computer-aided segmentation can save time and labor costs. It has been proved that Lagan is a robust model that can be applied to more medical physics network tasks (21). Not long ago, Hamabe et al. developed a rectal cancer segmentation software based on a U-Net deep neural network. They used this software for MRI image simulation analysis of 201 preoperative patients. It segmented the rectum, mesorectum, and tumors respectively, and eventually obtained DICE similarity coefficients (DSC) to artificial segmentation (rectum: DSC = 0.930, mesorectum: DSC = 0.917, tumor: DSC = 0.727) (80). Compared with MRI radiomics, which has been studied more in CRC, ultrasound is mainly used for early diagnosis of CRC. Song et al. designed a deep multi-view fusion network system based on endorectal ultrasonography (ERUS) to identify benign and malignant colorectal tumors, which could effectively reduce the workload of ultrasonic experts and the misdiagnosis rate (115).

Radiomics prediction of local advanced rectal cancer (LARC) after treatment is another crucial application. The ERUS can be used to identify early CRC, however, the diagnostic value for metastasis of advanced CRC is limited (113). The MRI examination after neoadjuvant treatment may be unreliable for pathologic complete response (PCR) identification (116). Interestingly, we can predict and stratify the risk of patients after neoadjuvant chemoradiotherapy (NCRT) by establishing a nomogram. This nomogram combines multi-parametric MRI information and clinicopathological factors, eliminating the effects of PCR intervention and helping to choose more accurate treatment options (77). Similarly, Farri et al. established an AI model based on the MRI image texture features to evaluate the PCR of 55 LARC patients after NCRT. The results showed that the AUC of 0.86, confirming that it is valuable in speculating PCR patients after NCRT (117). Radiomics is still widely used in the study of distal metastasis. Last year, Rocca et al. proposed to use the formal methods (FMS) combined with CT to monitor the liver metastasis of CRC. The overall accuracy rate reached 93.3%. The FMS appears to be trustworthy and valuable (74). There were also the combined models of radiomics, immunomics, and pathomics. They were beneficial in predicting lung metastasis of CRC (111).

## 4. The application of AI in CRC treatment

The treatments for CRC include surgical therapy, chemotherapy, targeted therapy, and other combined therapies. The application of AI in the treatment of CRC can design appropriate therapeutic plans for patients, providing patients with more personalized and precise medical decisions, and improving the prognosis. Common AI models for CRC treatment are summarized in Table 2.

**Table 2:** The summary of the application of AI in the treatment of CRC.

Theme	Year	Subject	Model	Sample	Results	Ref
Surgical treatment	2020	AI-assisted surgery	CNN	300 videos in operation	Accuracy = 81.0%; 83.2% (phase; action)	(118)
	2019	Robot-assisted surgery	da Vinci	Analyze from 206 RACRS patients	RM = 99.3%; 89.6% LN = 16 ± 6; 16 ± 8 LRR = 3.8%; 9.5% (colon; rectal)	(119)
	2021	AI-assisted LCRS	da Vinci	Analyze 600 images in 32 videos	DC = 0.84	(120)
	2022	Evaluate short outcomes	Senhance	Review outcomes in 55 Senhance assisted LCRC patients	Ileocecal resection = 32.7% high anterior resection = 20% D3 dissection = 74.5%	(121)
	2020	Automatic recognition	CNN	Recognize 71 Lap-S videos	Accuracy = 91.9%	(122)
	2021	Liver segment resection	da Vinci Xi	Present a video in a 54-year-old male patient	Operative time = 205 min estimated blood loss = 310 mL	(123)
	2020	Operation analysis	AIRAM	Test 25 ICG curve patterns	Processing time = 48.03 s	(24)
Chemoradiotherapy	2019	Assess therapy effect	RF	Assess performance from 55 patients	AUC = 0.86	(117)
	2022	Predict PCR after nCRT	RAPIDS	Study 933 patients	AUC = 0.812; sensitivity = 0.888 specificity = 0.740; NPV = 0.929 PPV = 0.512	(124)
	2021	Assess therapy effect	FFN/LR/SVM	Study 226 LARC patients	Accuracy = 0.67–0.75% AUC = 0.76–0.83% positive = 67–74%; NPV = 70–78% sensitivity = 68–79% specificity = 66–75%	(125)
	2018	Predict nCRT effect	DNN	Study 95 patients	Accuracy = 80%	(126)
	2020	Predict PCR after nCRT	ANN	Analyze 270 LARC patients	VSR = 1.57 (CEA levels)	(127)
	2022	Predict nCRT effect	MSCNN	Assess 150 WSI	AUC = 0.9337; 0.9091 (Camelyon; MSKCC)	(128)
	2019	Predict CRT response	CNN	Study 51 RC patients	AUC = 0.83	(129)
	2019	Predict nCRT effect	LR	Study 136 RC patients	AUC = 0.751; 0.831; 0.873 sensitivity = 66%; 71%; 75% specificity = 87.22%; 86.11%; 91.67% (pre-nCRT; early; combined)	(130)
	2020	Predict PCR,TRG, and NAR	LR	Collect and classify 132 nCRT and TME patients	AUC = 0.66; 0.80; 0.80 (NAR; PCR; TAG)	(131)
	2021	Predict and treat nCRT response	CFs-SVM	Analysis 428 patients	AUC = 0.834; 0.854 (training; validation)	(132)
Targeted therapy	2022	Identify therapy targets	MCODE	Extract four gene expression profile from database	Identify 8,931 DEGs in CRC patients	(133)
	2022	Design CAD approach	RF/SVP/CNN	Scanning 1,443 approved drugs	CAD design approach target p53 for treatment	(134)
	2022	Monitoring gene expression and drug effect	MLP	Study CRC cells genes phenomics	Mean accuracy = 9.48%↑(single track *VS* MLP)	(135)
	2021	Medicine precision	ML	Study STNs of CRC	The model with novel event freesurvival has a greater prediction	(136)
	2019	Tumor target segmentation	CAC-SPP	Evaluate two segmentation of tumor targets	DSC = 0.78 ± 0.08; 0.85 ± 0.03	(137)

AIRAM, artificial intelligence based real-time analysis microperfusion; AUC, area under the carve; ANN, artificial neural network; CNN, convolutional neutral network; CAD, computer aided drug; CAC, cascaded atrous convolution; DC, dice coefficient; DNN, deep neural network; DEGs, differential expressed genes; DSC, dice similarity coefficient; FFN, feedforward neural network; ICG, indocyanine green; LN, lymph nodes; LRR, locoregional recurrence rate; LCRS, laparoscopic colorectal surgery; Lap-s, laparoscopic sigmoidoscopy; LR, logistic regression; LARC, local advanced rectal cancer; MSCNN, multi-scale convolutional neural network; MCODE, molecular complex detection algorithm; MLP, machine learning phenomics; ML, machine learning; nCRT, neoadjuvant chemoradiotherapy; NPV, negative predictive value; NAR, neoadjuvant rectal score; PCR, pathological complete response; PPV, positive predictive value; RACRS, robot-assisted colorectal surgery; RM, radical margins; RF, random forest; RAPIDS, radiopathomics integrated prediction system; SVM, support vector machine; STNs, signal transduction network; SPP, spatial pyramid pooling; TRG, tumor regression grade; TME, total mesorectal excision; VSR, variable sensitivity ratio.

### 4.1. Surgical treatment

In recent years, with the development of minimally invasive surgery, the application of AI in the field of surgery has also been gradually valued. Compared with more complicated CRC surgery, AI was applied earlier in lung cancer and breast cancer surgery (138). Notably, people have begun to study the application of AI in colorectal surgery, and gradually realize that AI can provide a new direction for the development of colorectal surgery. Much research data on CRC is pouring out. Computer vision (CV) is a subfield of AI that can be used to analyze and evaluate video data (138). Some researchers in Japan have collected and analyzed 300 videos of laparoscopic colorectal surgery, hoping that these data sets may be employed to optimize the CNN performance in CV (118). South Korean scholars also conducted AI-based research on the perfusion of the indocyanine green (ICG) angiography system under laparoscopic colorectal surgery in the same year. They collected 200 ROIs from every 50 patients, a total of 10,000 ICG curves. And then they classified these data sets into 25 curve modes, confirming that the virtual microcirculation analysis system is more accurate with the assistance of AI (24).

Now, robotic surgery also achieves more remarkable development in the field of colorectal surgery, especially in rectal surgery. Even in the relatively difficult and complex transanal total mesorectal excision (taTME), which requires rich experience in laparoscopic operation, the robotic surgery has also been proved feasible (139). Compared with open surgery and laparoscopic surgery, robotic surgery has many advantages, such as shorter hospital stay, less perioperative bleeding, fewer complications, and improved postoperative quality of life. At the same time, it also can reduce the difficulty of the surgeon’s operation and relieve fatigue (140–142). In terms of long-term effects, the robotic surgery’s recurrence rate and mortality rate are comparable to that of laparoscopic surgery (143, 144). Recently, Igaki et al. successfully developed a flat image navigation system, which could be used to help surgeons identify anatomical tissue during TME. This system needs more image data to improve the accuracy of its recognition for future evaluation (120).

**Figure 5:**
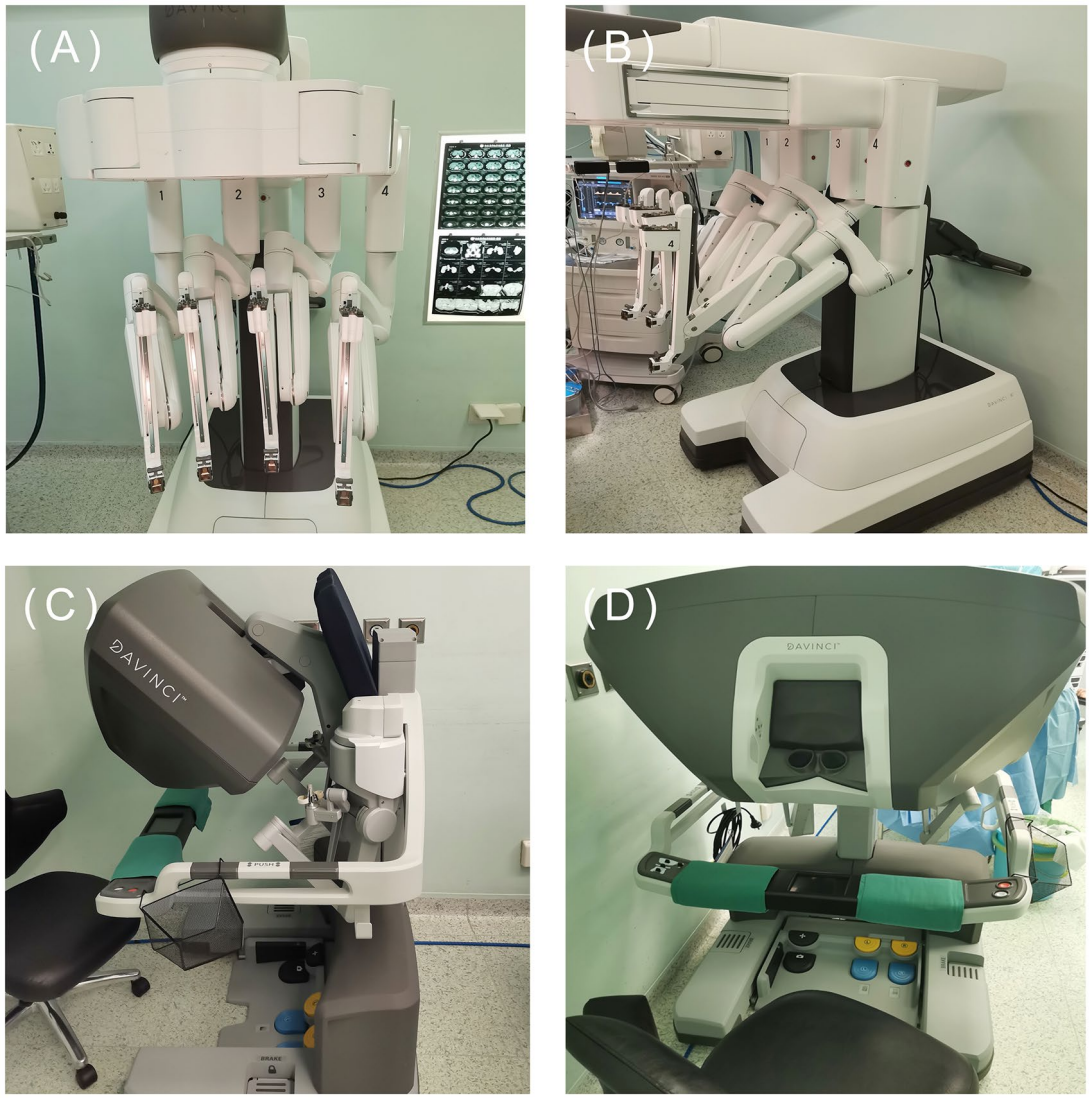
The fourth generation da Vinci. (Image source: The First Affiliated Hospital of Dalian Medical University.)

At present, the most widely used robot on the market is the da Vinci robot system, which has developed to the fourth generation (Figure 5). The fourth-generation robot has been dramatically improved in the cantilever system, with more subtle vision and precise operation, caring more about personalized needs. However, some problems remain, such as extended operation time, limited movement range, and poor sensory system (25, 145). The da Vinci SP system is a single-hole robot. It has only one robotic arm and three surgical instrument arms, which can be connected to three machines through one port. Experiments have confirmed that the da Vinci SP system is an excellent model for taTME and natural orifice specimen extraction (139, 145, 146). One of the biggest problems of robotic surgery is the high cost (145, 147). It indicates that the promotion of robotic surgery requires government financial support. With the progress of science and technology and establishment of a unified market, the cost of robotic surgery will also be reduced.

### 4.2. Chemoradiotherapy

NCRT is of great significance for CRC treatment, especially for patients with rectal cancer. Adjuvant chemotherapy is primarily used in the patients who are classified as intermediate risk (148). However, most patients do not need additional chemotherapy, so an accurate clinical decision-making is particularly important. The addition of AI is helpful for the treatment decision and efficacy evaluation of NCRT patients. In recent years, as one of the tools that can effectively reduce medical malpractice, AI-based clinical decision support systems (CDSSs) have attracted widespread attention. Experts in South Korea processed a chemotherapy recommender for CRC. This is the first CDSS in the country to reflect real data. It has satisfactory accuracy (AUC > 0.95), but the drawback is that the data source is relatively specific and single (149). Recently, kleppe et al. invented a DoMore-v1-CRC marker, developing the CDSSs based on DL to make a new risk division for patients after colectomy. When patients were classified as low risk, they could be exempted from NCRT. As a result, the survival rate of these patients improved significantly (148).

The prognosis evaluation of CRC patients is an important part for clinical doctors to choose appropriate treatment plans. DL-based assisted MRI can predict the metastasis of LARC patients receiving NCRT, which is a hot topic of current research (77, 124). Notably, Farrando et al. developed a classifier to predict the response of LARC patients who underwent NCRT. By evaluating the expression of lncRNAs, the researchers achieved satisfactory results (AUC = 0.93) (150). The researchers used biomarkers with significant stability, such as lncRNAs, in combination with available massive computational power to accurately predict drug resistance (151).

### 4.3. Targeted therapy

Targeted therapy is one of the effective methods for the treatment of CRC. Epithelial growth factor receptor (EGFR) is one of the vital drug targets. KRAS gene is highly sensitive to EGFR (152). However, the non-invasive prediction of the KRAS mutation state inCRC is considered as a significant challenge. Recently, some scholars have used the DL method based on a residual neural network to achieve this goal, attaining high predictive performance (AUC = 0.90) on the axis. This is helpful for further targeted treatment of CRC (153). The mutation rate of the BRAF gene in CRC can be as high as 10%. In another study, Beal et al. used a simpler RF data model to predict the V600E mutation in the BRAF (153). Many studies have supported that using AI to detect genetic mutations in CRC is a reliable way (151). These simple and cheap models will be the right choice for patients.

Abnormal mutations in genes and chromosomes can also cause drug resistance, which brings many obstacles to the treatment of CRC. In such an environment, the targeted therapy through drug delivery platform will contribute to precision medicine in the future (22, 151). Russo et al. used AI-based prediction model to analyze the patients who may have drug resistance before and after treatment. They achieved a sound effect (average AUC = 0.90) in the classification and targeted precision treatment (154). Interestingly, with the help of artificial algorithms, Hu et al. studied the competitive endogenous RNA (ceRNA) network about lncRNA and proposed 144 core genes for the first time, which could be used as target drugs for the treatment of CRC (155). The CRC research at the genetic scale can help us to understand the pathogenesis of tumors at the molecular level well, thus providing theoretical support for the diagnosis and treatment of CRC.

## 5. Discussion

Recently, the application of AI in CRC surgery has dabbled in various fields, and it has also achieved satisfactory results. There are indeed some challenges that we need to face and overcome. The development of AI needs to be supported by three elements: big data, computing power, and algorithm model. Today, the development of big data in China is still in the primary stage. More high-quality data is needed, and the interaction between data centers should be stronger. Without a large amount of high-quality data as the basis, even the most advanced algorithm model will not help. Therefore, we urgently need to standardize big medical data and increase interoperability among multiple centers (90, 147, 156). Moreover, there are specific problems in various imaging omics and AI models. For example, most models are based on retrospective data. The cases have strict admission and exclusion criteria. But there are more or fewer differences in the imaging standards of each center. Therefore, the repeatability of these models and the effectiveness of their application in the real world have yet to be fully evaluated (156).

Furthermore, the results obtained through the DL still lack interpretability. It cannot correctly judge the cause and give a reasonable explanation of the process and internal information of the algorithm. This phenomenon is called the “black box” problem in DL. Although there are still different opinions on the application of the black box in medicine, the transparency and interpretability of the algorithm have been taken as the core principles. It ensures that medical staff, patients, and other relevant personnel can fully understand clinical decisions, avoiding infringement of patients’ privacy, unclear responsibilities, and other ethical issues (156, 157). Now scholars have begun to study how the DL model makes decisions based on images and additional information, analyzing the causal relationship in its black box. Shao et al. used the DNN model to predict the survival rate of more than 20,000 patients within a year after major cardiovascular surgery. An impact score was defined innovatively to explain the results of the model prediction. The research on an interpretable DL is becoming more and more popular (158).

The development of AI in medicine still faces several limitations, and its application in colorectal surgery is also in its infancy. AI has indeed provided broad prospects for development in this field. This does not mean that AI can replace the role of clinicians. There is a need to strengthen cooperation between clinicians and computer experts to break through various transformation barriers. We should also evaluate clinicians’ acceptance of different AI systems and minimize the interference of AI in diagnosis and treatment.

## 6. Conclusion

With the continuous improvement of big clinical data, AI will develop rapidly in medicine. AI-based on various algorithms that combine with multiple medical imaging big data help to improve the early detection rate and diagnosis of CRC, conducting the early and systematic evaluation of patients. Moreover, it enhances the effect of adjuvant therapy, such as NCRT and targeted treatment, strengthening patients’ prognosis monitoring. Through continuous optimization and development, AI will make greater contributions to the diagnosis and treatment of CRC in the era of precision medicine.

## Author contributions

ZY wrote the paper. CY and LZ performed the revision and approval of the final version. LZ and ZY performed literature research. SQ corrected the writing of the paper. All authors contributed to the article and approved the submitted version.

## Conflict of interest

The authors declare that the research was conducted in the absence of any commercial or financial relationships that could be construed as a potential conflict of interest.

## Publisher’s note

All claims expressed in this article are solely those of the authors and do not necessarily represent those of their affiliated organizations, or those of the publisher, the editors and the reviewers. Any product that may be evaluated in this article, or claim that may be made by its manufacturer, is not guaranteed or endorsed by the publisher.
